# Free Fatty Acid-Induced PP2A Hyperactivity Selectively Impairs Hepatic Insulin Action on Glucose Metabolism

**DOI:** 10.1371/journal.pone.0027424

**Published:** 2011-11-07

**Authors:** Thomas Galbo, Grith Skytte Olsen, Bjørn Quistorff, Erica Nishimura

**Affiliations:** 1 Diabetes Research Unit, Department of Insulin Biology, Novo Nordisk A/S, Måløv, Denmark; 2 Department of Biomedical Science, The Panum Institute, University of Copenhagen, Copenhagen, Denmark; Universita Magna-Graecia di Catanzaro, Italy

## Abstract

In type 2 Diabetes (T2D) free fatty acids (FFAs) in plasma are increased and hepatic insulin resistance is “selective”, in the sense that the insulin-mediated decrease of glucose production is blunted while insulin's effect on stimulating lipogenesis is maintained. We investigated the molecular mechanisms underlying this pathogenic paradox. Primary rat hepatocytes were exposed to palmitate for twenty hours. To establish the physiological relevance of the *in vitro* findings, we also studied insulin-resistant Zucker Diabetic Fatty (ZDF) rats. While insulin-receptor phosphorylation was unaffected, activation of Akt and inactivation of the downstream targets Glycogen synthase kinase 3α (Gsk3α and Forkhead box O1 (FoxO1) was inhibited in palmitate-exposed cells. Accordingly, dose-response curves for insulin-mediated suppression of the FoxO1-induced gluconeogenic genes and for *de novo* glucose production were right shifted, and insulin-stimulated glucose oxidation and glycogen synthesis were impaired. In contrast, similar to findings in human T2D, the ability of insulin to induce triglyceride (TG) accumulation and transcription of the enzymes that catalyze *de novo* lipogenesis and TG assembly was unaffected. Insulin-induction of these genes could, however, be blocked by inhibition of the atypical PKCs (aPKCs). The activity of the Akt-inactivating Protein Phosphatase 2A (PP2A) was increased in the insulin-resistant cells. Furthermore, inhibition of PP2A by specific inhibitors increased insulin-stimulated activation of Akt and phosphorylation of FoxO1 and Gsk3α. Finally, PP2A mRNA levels were increased in liver, muscle and adipose tissue, while PP2A activity was increased in liver and muscle tissue in insulin-resistant ZDF rats. In conclusion, our findings indicate that FFAs may cause a selective impairment of insulin action upon hepatic glucose metabolism by increasing PP2A activity.

## Introduction

Insulin-resistance has been recognized for decades as a hallmark of T2D, yet the molecular mechanisms underlying this condition has been, to a great extent, elusive. T2D is characterized by the classic triad of hyperinsulinemia, hyperglycemia and hypertriglyceridemia with the presence of hyperglycemia in the face of hyperinsulinemia being the definition of insulin resistance. The hyperglycemia in T2D is in part due to an increased rate of hepatic glucose output. This increase is partially explained by a resistance to the ability of insulin to suppress hepatic gluconeogenesis and stimulate net glycogen synthesis [1], [2].

*Hepatic* insulin resistance has been closely examined in the LIRKO (liver-insulin receptor knock-out) mouse as this represents the ultimate model of insulin resistance in the liver. This mouse displays hyperglycemia and hyperinsulinemia similar to what is observed in T2D in man, suggesting that hepatic insulin resistance contributes to the development of T2D. However, contrary to what is observed in human T2D, LIRKO mice have low plasma TG and low hepatic TG content [3]. This discrepancy highlights the paradox, that in T2D, hepatic insulin resistance seems to be “selective” with only some actions of insulin being blunted in the liver - resulting in the inability of insulin to suppress hepatic glucose production, while the effect of insulin in inducing hepatic lipogenesis is preserved [4].

In the liver, insulin acts to stimulate phosphorylation and activation of Akt which in turn phosphorylates and inactivates the transcription factor FoxO1, which induces gluconeogenesis under fasting conditions [5], [6], [7]. Akt also phosphorylates and inactivates Gsk3α in response to insulin [8], and since Gsk3α normally inhibits Glycogen Synthase (GS), insulin stimulates GS activity and glycogen synthesis. Meanwhile, insulin acts through atypical PKCs (aPKCs) to stimulate transcription and activation of the transcription factor Sterol regulatory element-binding protein 1c (Srebp1c) [9] which then activates the transcription of several enzymes involved in fatty acid and TG synthesis [10]. In studies of animal models of insulin resistance, i.e. ob/ob mice and Gato-Kakazaki rats, focusing on hepatic Akt and aPKCs, insulin-activation of Akt but not aPKCs was impaired [11], [12].

Insulin receptor downstream signalling has been studied extensively in attempts to identify the molecular alterations underlying the defective insulin-stimulated Akt activation in T2D, and an increasingly complex signalling network is appearing [13]. Still, the mechanism by which Akt is *in*activated and how this relates to metabolic diseases is not well understood. However, five different phosphatases (PP2A, PTEN, Calcineurin and Phlpp1&2) have recently been shown to be involved in dephosphorylation and inactivation of Akt [14].

FFA levels in plasma are increased in T2D [15]. Furthermore, *in vitro* FFA exposure has been shown to induce insulin resistance by Akt-activation in liver cells [16]. Because FFA-induced insulin resistance may play a key role in T2D, we have carried out a comprehensive study of the effects of FFA on the molecular mechanisms involved in insulin-resistance, insulin-regulated glucose and fat metabolism in rat liver cells *in vitro*. We hypothesized that FFA-induced insulin-resistance to Akt activation could be mediated by excessive activity of Akt-inactivating phosphatases and, not as previously assumed, a consequence of impaired downstream insulin receptor signalling. Finally, we sought to establish the relevance of our *in* vitro findings through studies of insulin-resistant ZDF rats.

We found that the inhibitory effect of FFAs upon insulin-stimulated activation of hepatic Akt and its action upon glucose metabolism may indeed be the result of excessive PP2A activity, and that PP2A is also hyperactive in an animal model of insulin resistance.

## Materials and Methods

### Cell Culture

Hepatocytes were isolated using a standard collagenase-perfusion protocol [17] from male Sprague-Dawley rats (8 weeks old, 200–300 g). The hepatocytes were cultured in medium 199, 5.5 mM glucose + GlutaMAX (Gibco) +0.5% Human Serum Albumin (HSA) (Sigma) +100 nM Decadron (MSD) +1% Penicillin/Streptomycin (Gibco). Cells were rendered insulin-resistant by addition of 0.5 mM palmitate (Sigma) to the medium for twenty hours. Control cells were grown for the same time, in the same medium albeit without addition of palmitate. Experiments on insulin-resistant cells were performed in the presence of 0.5 mM palmitate.

### Real-time PCR

RNA was isolated from cells/tissue using the RNeasy Mini Kit (Qiagen). cDNA was synthesized using the iScript kit (Bio-Rad). Primer-probesets were from TaqMan Gene Expression Assays (Applied Biosystems) (except Srebp1c) and PCR reactions were performed using a TaqMan Master mix (Applied Biosystems) and a MX3000P system (Agilent). Srebp1c custom primer-probesets were purchased from Applied Biosystems with the sequences: Forward: CGCTACCGTTCCTCTATCAATGAC; Reverse: AGTTTCTGGTTGCTGTGCTGTAAG; Probe: GTGGTGGGCACTGAGGC. Ct values were normalized to those of Ppib.

### Western Blotting

Cellular protein lysates were assayed with antibodies against Akt, pAkt (Ser473), FoxO1, pFoxO1 (Ser256), Gsk3α, pGsk3α(Ser21), IRβ, paPKC (Thr410/403) (Cell Signalling Technology) meth-PP2AC, phopho-Tyr 4G10 (Millipore) PP2Aα, α-Calcineurin (Sigma) Phlpp1&2 (Bethyl) Pten (Transduction Labs), β-Actin (Abcam) aPKC and Srebp1 (2A4) (Scbt). Secondary antibodies were horseradish peroxidise-coupled and ECL reagent (BioVision) was used for detection. Quantification was performed using ImageGuage 4.0 (Fujifilm).

### TG accumulation

After 24 h of insulin stimulation, cellular TG levels were determined using the Serum Triglyceride Determination Kit (Sigma) according to manufacturer's instructions.

### Glycogen accumulation

After 24 h of insulin stimulation, cellular glycogen was digested with Amyloglucosidase (Sigma) for 3 h (37°C). Glucose was measured on aliquots of the lysate in a hexokinase/Glu6PDH assay previously described [18].

### Gluconeogenesis

Hepatocytes were given medium 199 without glucose (Gibco) supplemented with 2 mM pyruvate (Sigma). After 24 h of insulin-stimulation, glucose contained in the medium was measured as above. Cells contained only minimal amounts of glycogen before stimulation so any contribution of glycogenolysis was negligible.

### Glucose disposal

Hepatocytes were challenged with medium 199 (Gibco) containing 15.5 mM glucose. After 24 h of insulin-stimulation, glucose contained in the medium was measured as above.

### PP2A activity

PP2A activity was measured in 500 ug palmitate-treated, control hepatocytes or animal tissues using the PP2A immunoprecipitation phosphatase assay kit (Millipore) according to the manufacturer's instructions.

### Experimental animal studies

Eight fa/fa rats (∼500 g) and six Fa/Fa rats (∼300 g) (male, 13–15 weeks, Charles River) fed a normal *ad libitum* chow diet (Altromin 1320) were fasted overnight before sacrifice. Tissues (liver, red quadriceps muscle and ependidymal fat) were freeze-clamped in liquid nitrogen and kept frozen until analysis. All animal studies were approved by and carried out in accordance with the Danish Justice Department guidelines (Animal Experiment Inspectorate) (permit no. BidR 192000/561-332).

### Chemicals

Myristylated aPKC pseudosubstrate (Calbiochem), Triciribine (Cayman), Cantharidin and Okadaic acid (Sigma), NPH insulin (Novo Nordisk).

### Statistical analysis

Statistical significance was calculated using an unpaired Student's t-test.

## Results

### Insulin receptor phosphorylation is retained, but Akt activation is impaired in cultured primary rat hepatocytes treated with palmitate

**Figure 1:**
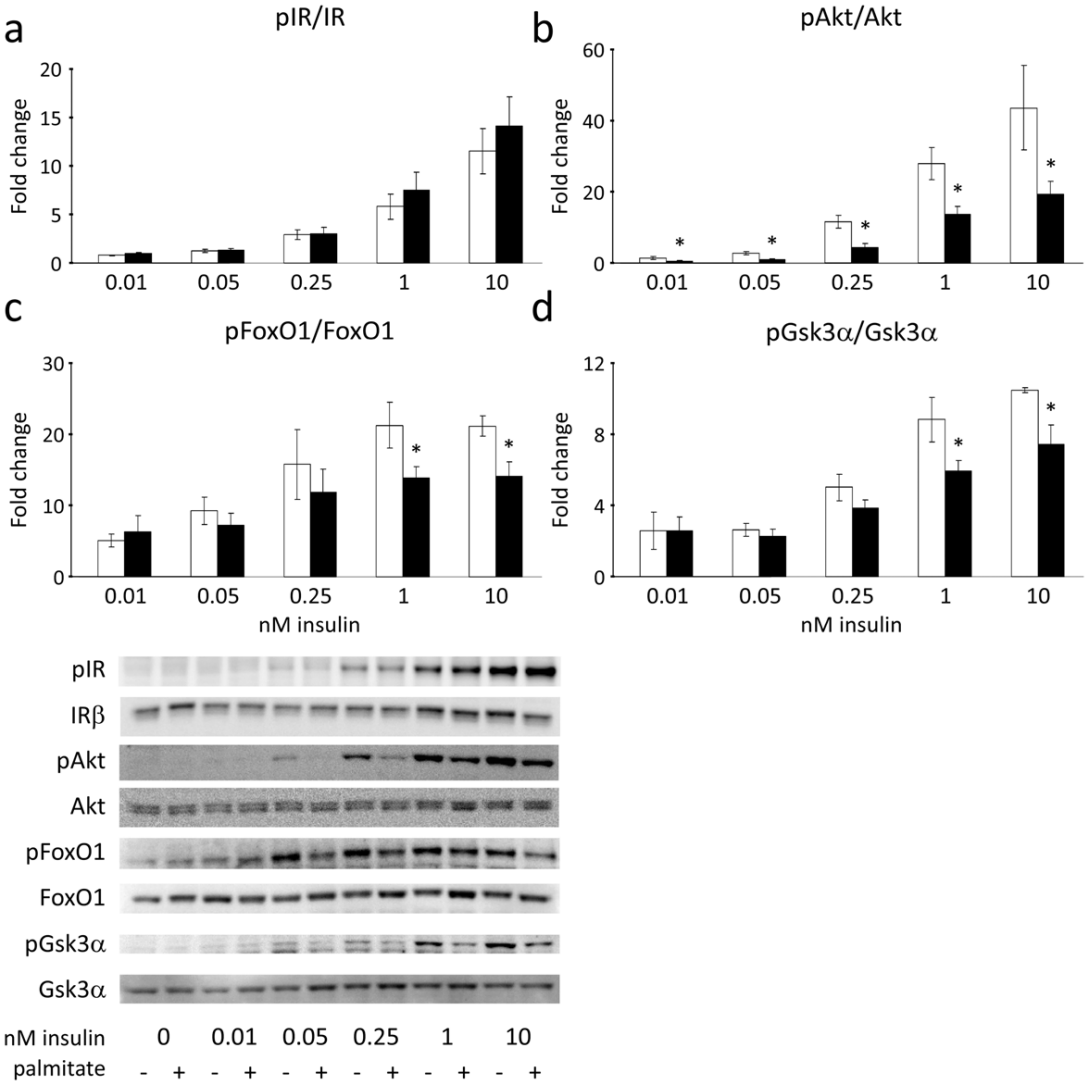
Palmitate does not affect insulin-receptor phosphorylation, but impairs Akt activation. □ Control; ▪ 0.5 mM Palmitate. Palmitate has no effect on insulin receptor phosphorylation, but Akt activation is impaired in cultured primary rat hepatocytes treated for 20 h with palmitate. (**a**) The insulin-stimulated insulin receptor phosphorylation to total insulin receptor ratio is not altered in hepatocytes treated with palmitate. (**b**) However, insulin-stimulated phosphorylation of Akt Ser473 is significantly inhibited by palmitate. This effect is conferred onto the Akt substrates FoxO1 (Ser256) (**c**) and Gsk3α (Ser21) (**d**) which both show decreased insulin-stimulated phosphorylation. Fold change is relative to no insulin. Phosphorylation levels of individual proteins were normalized to the total amounts of the respective proteins. Data are averages of western blot quantifications +/− std. error of the mean, n = 8. * indicates p<0.05 between groups. Representative western blots are shown.

In hepatocytes treated for twenty hours with 0.5 mM palmitate, insulin-stimulated (15 min) insulin receptor (IR) phosphorylation was maintained in hepatocytes exposed to palmitate (Fig. 1a) compared to insulin-stimulated control cells. However, insulin-stimulated phosphorylation (Ser473) and activation of Akt was significantly impaired (Fig. 1b) and, correspondingly, a reduced phosphorylation of the Akt substrates FoxO1 (Ser256) (Fig. 1c) and Gsk3α (Ser21) (Fig. 1d) was observed in response to insulin. In contrast, no difference was detected in the effect of insulin upon phosphorylation of aPKC (Thr410/403) (Supporting Information S1).

### Palmitate-induced insulin resistance is characterized by continued insulin action on aPKC-regulated lipogenic genes and impaired Akt-mediated gene regulation

**Figure 2:**
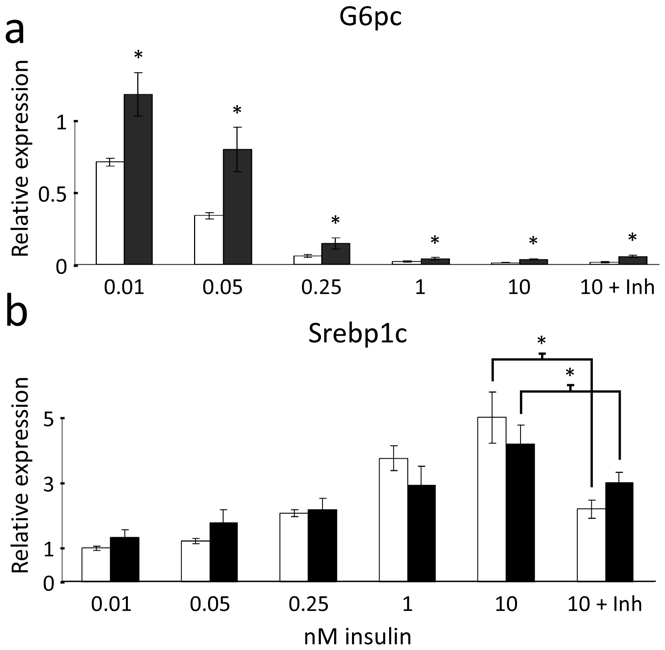
Palmitate-induced insulin resistance results in impaired G6pc suppression and continued action on aPKC regulated Srebp1c. □ Control; ▪ 0.5 mM Palmitate. (**a**) Insulin-suppression of G6pc mRNA was significantly inhibited in hepatocytes treated for 20 h with palmitate *in vitro*. This suppression was not affected by preincubation with 20 µM of a pseudosubstrate inhibitor of the aPKCs (Inh). (**b**) On the other hand, insulin-stimulated induction of Srebp1c mRNA was maintained in palmitate-treated cells. Induction could, however, be inhibited by addition of the aPKC inhibitor (Inh). Relative expression is relative to no insulin. Data are averages of real-time PCR results +/− std. error of the mean, n = 10. * indicates p<0.05 between groups.

Hepatic gluconeogenesis is regulated by the rate-limiting enzymes Glucose-6-phosphatase (G6pc) and Phosphoenolpyruvate carboxykinase 1 (Pepck) [19], [20] which are targets of FoxO1. Since insulin-action on Akt, and in turn inactivation of FoxO1, was impaired in hepatocytes treated with palmitate, insulin's ability to suppress transcription of G6pc and Pepck mRNA was investigated. Treatment with palmitate resulted in a significant decrease in insulin's ability (6 h) to suppress G6pc and Pepck mRNA levels (Fig. 2a, and Supporting Information S2). Interestingly, insulin was still able to induce the transcription of the aPKC-controlled master regulator of lipogenesis, Srebp1c (Fig. 2b). Insulin-stimulated induction of the Srebp1c target genes Fatty acid synthase (Fasn), Acetyl-CoA carboxylase (Acc), Glucokinase (Gck) and mitochondrial Glycerol-3-phosphate acyltransferase (Gpam) as well as Diacylglycerol acyltransferase-2 (Dgat2) was also maintained (Supporting Information S3). The induction of Srebp1c could, however, be blocked by treating the hepatocytes with 20 µM of a myristylated pseudosubstrate inhibitor of the aPKCs (Fig. 2b) which does not inhibit Akt activity [21]. Incubation of unstimulated hepatocytes with the pseudosubstrate alone resulted in a lowering of baseline Srebp1c levels, likely reflecting a suppression of basal aPKC activity (not shown). In contrast, this inhibitor did not affect insulin-induced suppresion of G6pc (Fig. 2a), whereas 1 µM of a specific Akt inhibitor, which does not affect PKC activity [22], (Triciribine (TCN)) impaired insulin suppression of G6pc (Supporting Information S4) supporting the notion that insulin suppression of G6pc is regulated by Akt, while aPKCs mediate the induction of lipogenic genes.

### Palmitate impairs insulin's action on Akt-dependent processes, but does not affect insulin-stimulated TG accumulation

**Figure 3:**
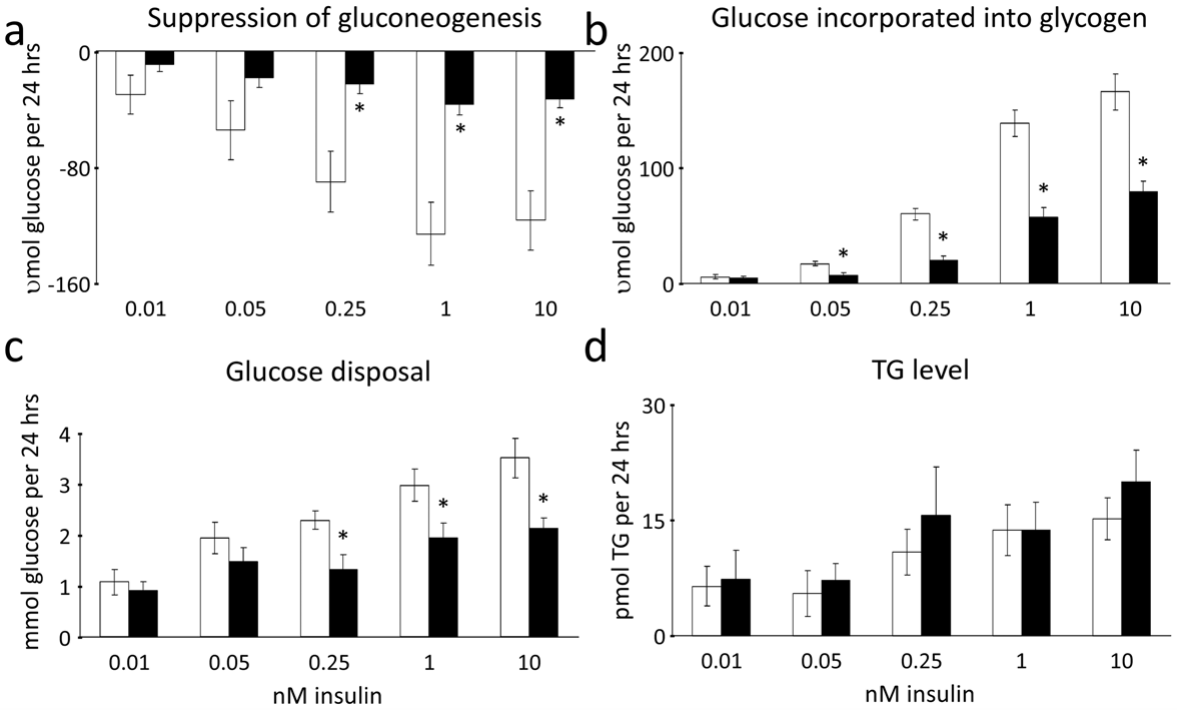
Palmitate-induced insulin resistance reflects an impairment of insulin action on Akt-dependent processes. □ Control; ▪ 0.5 mM Palmitate. However, insulin action on TG accumulation is maintained. (**a**) Insulin-stimulated suppression of gluconeogenesis (from medium containing 2 mM pyruvate), (**b**) incorporation of glucose into glycogen (from 5.5 mM glucose) and (**c**) glucose disposal (from 15.5 mM glucose) were significantly inhibited in hepatocytes treated for 20 h with palmitate *in vitro*. Meanwhile, insulin-stimulated TG accumulation was maintained in palmitate-treated hepatocytes (**d**). Results are expressed as differences from the rates in cells given no insulin. Data are averages of 8–10 independent experiments +/− std. error of the mean. * indicates p<0.05 between groups.

To examine how the observed selective insulin-resistance in signalling and gene expression translated into functional differences, biological endpoints of insulin signalling, gluconeogenesis, glycogen synthesis, glucose disposal and TG synthesis, were determined. Consistent with impaired insulin stimulated Akt activation, FoxO1 inactivation and gluconeogenic gene expression, it was found that insulin-suppression of gluconeogenesis (from 2 mM pyruvate) was similarly impaired in hepatocytes treated with palmitate (Fig. 3a). Likewise, insulin-stimulated glycogen synthesis (from 5.5 mM glucose) and general glucose disposal (when challenged with 15.5 mM glucose) (Fig. 3b and 3c) were also attenuated. However, in line with the unaltered effects of palmitate on Srebp1c regulated gene-expression patterns, insulin-stimulated accumulation of TGs was maintained in the insulin-resistant cells (Fig. 3d).

### Activity of the Akt-inactivating phosphatase PP2A is increased in insulin-resistant hepatocytes

**Figure 4:**
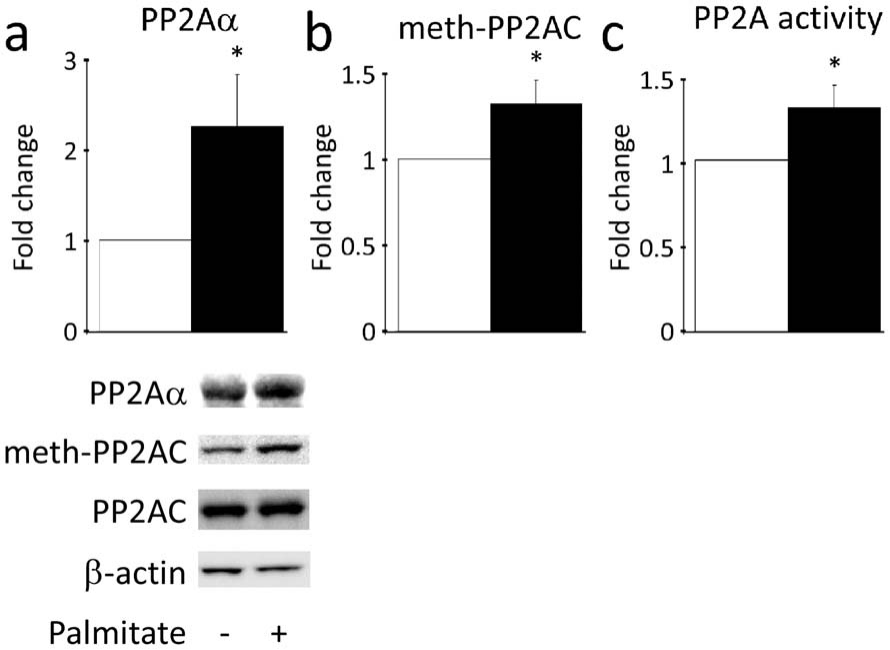
PP2A activity is increased in hepatocytes treated with palmitate. □ Control; ▪ 0.5 mM Palmitate. The protein level of a PP2A structural subunit (PP2Aα) was upregulated in hepatocytes treated with palmitate (**a**). Meanwhile, PP2A activity was increased by approximately 30 per cent in insulin-resistant palmitate-treated hepatocytes (**b, c**). PP2A-activity was estimated by the degree of methylation of the PP2A catalytic subunit (**b**) or a direct phosphatase activity assay (**c**). Protein levels were normalized to β–actin. Data are averages of western blot quantifications or PP2A activity measurements +/− std. error of the mean, n = 10. * indicates p<0.05. Representative western blots are shown for **a** and **b**.

The effect of any signalling molecule is dependent on the balance between its activation and its inactivation. Thus, due to the observations indicating that impairment of Akt activity is a key phenomenon in lipid mediated hepatic insulin resistance the role of phosphatases in this process was examined. Five different phosphatases are known to be involved in inactivation of Akt [23], so therefore, potential differences in the activity of these phosphatases in hepatocytes rendered insulin-resistant with palmitate, compared to control cells, were monitored. While the protein levels of Pten, α-Calcineurin and Phlpp1&2 were not different from control cells (Supporting Information S5), a structural subunit of PP2A (PP2Aα) was found to be upregulated 2.2-fold (Fig. 4a). To determine whether the activity of PP2A was increased, methylation status of the catalytic subunit of PP2A was determined. Methylation of this particular subunit is required for the assembly and activation of the PP2A holoenzyme [24], [25]. PP2A activity was also evaluated using an immunoprecipitation activity assay. Both assays suggested that PP2A activity was significantly increased by approximately 30 per cent (Fig. 4b, c) in palmitate-treated hepatocytes, suggesting that Akt is inactivated by PP2A at an accelerated rate.

### Inhibition of PP2A activity results in an increased insulin-stimulated activation of Akt and phosphorylation of its downstream substrates

**Figure 5:**
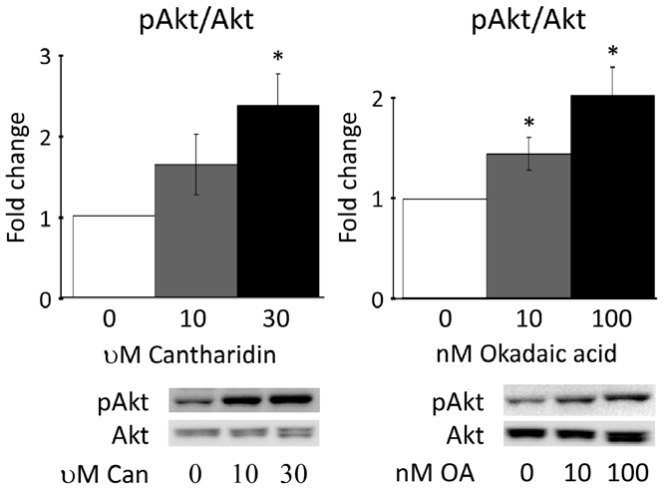
Inhibition of PP2A activity results in an increased insulin-stimulated activation of Akt. Cantharidin (Can) (**a**) or Okadaic acid (OA) (**b**) inhibition of PP2A resulted in an increased Ser473 phosphorylation of Akt in palmitate-treated hepatocytes stimulated with 10 nM insulin. Fold change is relative to no Cantharidin or Okadaic acid. Akt phosphorylation levels of were normalized to the total amounts of Akt. Data are averages of western blot quantifications +/− std. error of the mean, n = 8. * indicates p<0.05 vs. no Cantharidin or Okadaic acid. Representative western blots are shown.

To confirm the relevance of increased PP2A activity for Akt activation in hepatocytes two specific PP2A inhibitors, Cantharidin [26], [27] and Okadaic acid [28], were used. Incubation of palmitate-treated hepatocytes with Cantharidin or Okadaic acid for thirty min prior to stimulation with 10 nM insulin (15 min) resulted in a significantly increased activation of Akt (Fig. 5a, b). This increased activation of Akt was propagated onto FoxO1 and Gsk3α (results not shown) which were both phosphorylated and inactivated to an increased extent. Of further interest, in other unpublished studies, we have found that PP2A inhibition neither affects the tyrosine phosphorylation state of the insulin receptor nor aPKC Thr410/403 phosphorylation (data not shown).

### PP2A mRNA levels are upregulated in liver, muscle and fat tissues, while PP2A activity is increased in liver and muscle in severely insulin-resistant ZDF rats

**Figure 6:**
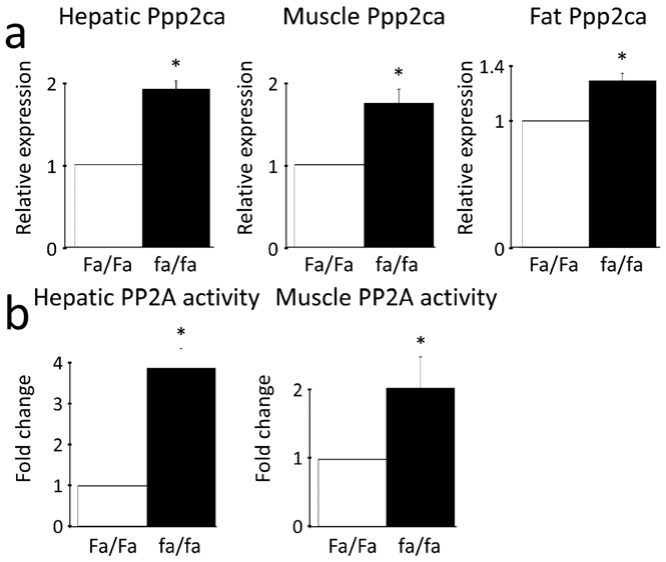
The Akt-inactivating phosphatase PP2A is hyperactive in insulin-resistant ZDF rats. PP2A levels were upregulated in all major insulin target tissues while PP2A activity was upregulated in liver and muscle in severely insulin-resistant ZDF rats. (**a**) Hepatic, muscle and fat mRNA levels of the catalytic subunit of PP2A (Ppp2ca) and (**b**) hepatic and muscle PP2A activity were found to be significantly elevated in obese fa/fa ZDF rats compared to their lean Fa/Fa counterparts. Relative expression and fold change is relative to Fa/Fa expression or activity levels. Data are averages of real-time PCR results or PP2A activity measurements +/− std. error of the mean. n = 8 for fa/fa rats and n = 6 for Fa/Fa rats. * indicates p<0.05.

**Figure 7:**
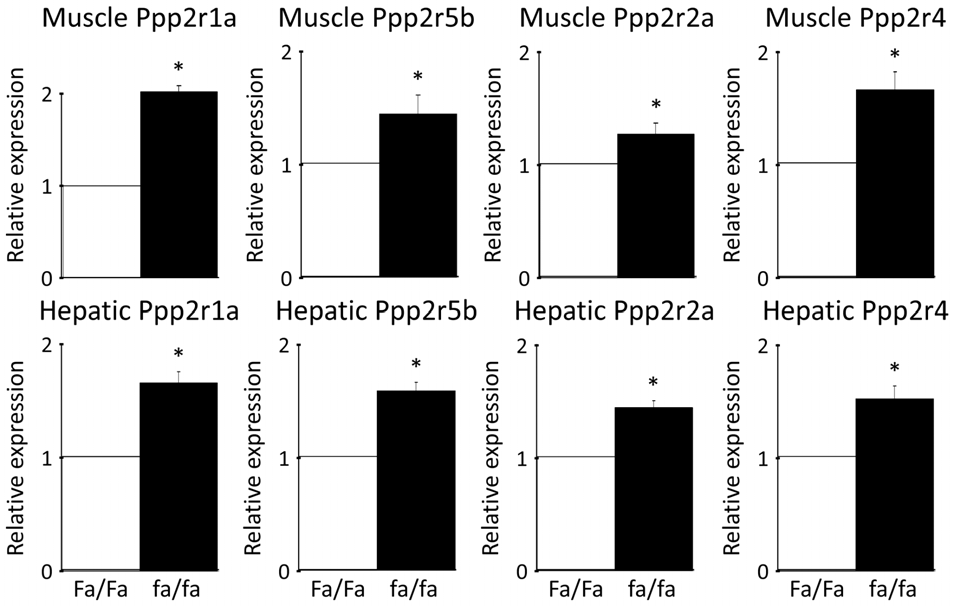
Several PP2A subunits are upregulated in ZDF rats. The PP2A subunits PP2Aα (Ppp2r1a), B56β (Ppp2r5b), B55α (Ppp2r2a) and Ptpa (Ppp2r4) were significantly upregulated in both liver and muscle of fa/fa rats compared to their lean Fa/Fa counterparts. Relative expression is relative to Fa/Fa expression levels. Data are averages of real-time PCR results +/− std. error of the mean. n = 8 for fa/fa rats and n = 6 for Fa/Fa rats. * indicates p<0.05.

The leptin-deficient (fa/fa), hyperphagic Zucker Diabetic Fatty rat develops overt obesity and severe hepatic and peripheral insulin resistance and is a commonly used animal model of T2D [29], [30]. To establish the physiological relevance of the *in vitro* findings, differences in phosphatase levels between Zucker obese (fa/fa) and lean rats (Fa/Fa) fed a normal diet were examined. fa/fa rats were severely insulin-resistant, hyperglycaemic and hypertriglyceridemic, and had higher levels of plasma free fatty acids than their lean (Fa/Fa) counterparts (Supporting Information S6). The mRNA levels of the catalytic PP2A subunit (Ppp2ca) were significantly elevated in liver, muscle (red quadriceps) and fat (ependidymal) tissues in the fa/fa rats (Fig. 6a) while PP2A activity was also increased in both fa/fa liver and muscle tissue (Fig. 6b). Similarly, the structural PP2Aα (Ppp2r1a) as well as the regulatory B56β (Ppp2r5b), B55α (Ppp2r2a) and Ptpa (Ppp2r4) subunits were significantly upregulated in both liver and muscle (Fig. 7), but not in fat (not shown). The expression levels of the other known Akt-inactivating phosphatases Phlpp1&2, Pten and Calcineurin were also examined in liver, muscle and fat. We found that α-Calcineurin (Ppp3ca) was significantly upregulated in fa/fa livers, while α-Calcineurin and Phlpp1 were significantly upregulated in fa/fa muscle (Supporting Information S7). This strongly suggests that phosphatase activity, and in particular PP2A activity, may play a crucial role in the defective activation of Akt and the molecular pathogenesis of insulin resistance *in vivo*.

## Discussion

In the present study, we have demonstrated that palmitate-induced insulin resistance in primary rat hepatocytes is a selective phenomenon similar to what is observed in T2D in humans in the sense that the effects of insulin upon glucose metabolism are blunted, while the insulin effect on lipogenesis and TG accumulation is unchanged. This phenomenon is in contrast to what is observed in LIRKO mice, where all the effects of insulin are blunted [3], [4]. The differential impairment of insulin effects in palmitate exposed hepatocytes is explained by the fact that insulin is limited in its ability to suppress the transcription of gluconeogenic genes, but maintains its ability to induce transcription of Srebp1c and lipogenic genes. Akt normally mediates insulin inhibition of the key gluconeogenesis-inducing transcription factor FoxO1 [6], while aPKCs mediate insulin-stimulated lipogenesis through Srebp1c [9]. We have shown that palmitate treatment of hepatocytes inhibits insulin-stimulated Akt phosphorylation explaining the inhibited action upon FoxO1, Gsk3α and, in turn, gluconeogenesis and glycogen synthesis. In contrast, we have shown that palmitate does not influence the stimulation by insulin of aPKC-mediated lipogenic gene expression and that aPKCs do not play a role in insulin action upon gluconeogenic gene expression. These findings indicate that the selectivity of lipid-induced insulin resistance may be explained by Akt activation being defective and aPKC activation being maintained. This complements previous findings in livers of diabetic animals.

Our data also indicate that the activity of Akt-inactivating phosphatase PP2A [31] is increased in palmitate treated hepatocytes. PP2A has previously been shown to be activated by palmitate [32] and to inhibit insulin-stimulated Akt activity, phosphorylation at Ser473 and glycogen synthesis in C2C12 myotubes [33]. In 3T3-L1 preadipocytes, PP2A has been shown to directly inhibit Akt activity and Ser473 phosphorylation by two distinct mechanisms – without affecting the immediate upstream Akt activator PDK1 [34]. We showed that inhibition of PP2A ameliorates palmitate-induced inhibition of Akt activity and restores Akt action on its substrates *in vitro*. Compatible with a potentially marked patophysiological relevance of PP2A, comparison between Figs. 1 and 5 shows that the impairment of Akt activity by FFA could be fully abolished by the PP2A inhibition. Yet, further studies applying genetic silencing of PP2A will be important to validate the effects observed using the inhibitors. It is of note that PP2A activity was upregulated in liver and muscle while PP2A mRNA was upregulated in all major insulin target tissues in diabetic ZDF rats. In fact, in liver and muscle the catalytic subunit, the structural PP2Aα subunit as well as the regulatory Ptpa, B56β and B55α subunits were all elevated. Regulatory PP2A subunits are believed to control PP2A activity and provide its substrate specificity. Accordingly, the upregulation of Ptpa, B56β and B55α is particularly interesting as Ptpa has been shown to be required for PP2A activation [35], [36] while B56β [37] and B55α [38] have both been shown to target PP2A to Akt, thus potentially explaining why insulin activation of Akt is blunted while the effect of insulin upon lipogenesis is maintained. Previous studies have indicated that insulin-resistance in other tissues than liver may also reflect a selective impairment of Akt activation, while e.g. phosphorylation of MAPKs is not impaired [39]. Because the Ras-MAPK pathway is mainly believed to be involved in the cell growth, differentiation and proliferation effects of insulin, and not the metabolic effects of insulin [13], we did not include measurement of the phosphorylation state of MAPKs in the present study of hepatic glucose- and lipid metabolism. We assessed phosphorylation of the Ser473 residue of Akt. However, as overexpression of PP2A – albeit only a single regulatory subunit and in more primitive cells (FL5.12 and NIH3T3) [40] – has suggested that PP2A may be more specific for the Thr308 residue, evaluation of the phosphorylation levels of Thr308 would also be of interest.

Among the other four known Akt-inactivating phosphatases, α-Calcineurin mRNA was found to be elevated both in fa/fa liver and muscle tissues while Phlpp1, in accordance with previous findings in T2D patients, was upregulated in muscle [41].

Plasma FFAs are elevated in obesity and T2D [15], and lowering FFA levels increases insulin sensitivity [42], while raising FFAs increases insulin resistance [43].

**Figure 8:**
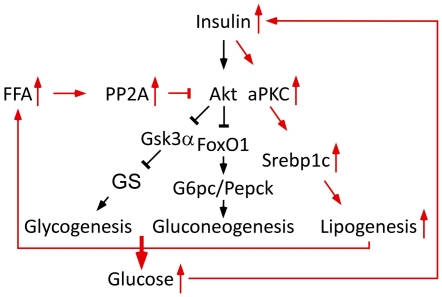
Proposed model for selective insulin resistance. Black arrows designate physiological conditions, whereas red arrows designate altered conditions in insulin resistance. For further explanation, see text.

Based on the collected findings presented here, we propose a model (Fig. 8) in which obesity is accompanied by increased FFA levels which activate PP2A and, in turn, elicits its inhibitory effect upon Akt. Decreased insulin-stimulated Akt activation results in an impaired ability of insulin to suppress hepatic glucose output. The ensuing hyperglycemia stimulates insulin secretion. However, as insulin maintains its ability to drive lipogenesis through aPKCs and Srebp1c, elevated systemic insulin levels lead to an increased rate of lipogenesis. This creates a vicious cycle, in which insulin resistance leads to accelerated lipogenesis and, in turn, more insulin resistance and an elevated risk of atherosclerosis and other devastating complications of dyslipidemia.

In conclusion, our findings indicate that FFAs cause hepatic insulin resistance by increasing PP2A activity, which impairs Akt-mediated gene regulation. This insulin resistance is selective in the sense that it does not affect insulin action on aPKC-regulated lipogenic genes. In turn, this highlights the need for more studies into the roles of phosphatases in metabolic disease, as well as the therapeutic potential of phosphatase inhibition.

## Supporting Information

Supporting Information S1 **Palmitate has no effect on insulin-stimulated aPKC phosphorylation in cultured primary rat hepatocytes.** □ Control; ▪ 0.5 mM Palmitate. Fold change is relative to no insulin. The phosphorylation level was normalized to the total amounts of aPKC. Data are averages of western blot quantifications +/− std. error of the mean, n = 8. * indicates p<0.05 between groups. Representative western blot is shown.
(TIF)

Supporting Information S2 **Palmitate-induced insulin resistance is characterized by impaired Pepck suppression.** □ Control; ▪ 0.5 mM Palmitate. Insulin-suppression of Pepck mRNA was significantly inhibited in hepatocytes treated for 20 h with palmitate *in vitro*. Relative expression is relative to no insulin. Data are averages of real-time PCR results +/− std. error of the mean, n = 10. * indicates p<0.05 between groups.
(TIF)

Supporting Information S3 **Insulin-stimulated induction of lipogenic Srebp1c target genes was maintained in palmitate-treated cells.** □ Control; ▪ 0.5 mM Palmitate. In contrast to the gluconeogenic genes, G6pc and Pepck, insulin maintained its effect upon Dgat2, Fasn, Acc, Gck and Gpam expression in insulin-resistant, palmitate-treated cells. Relative expression is relative to no insulin. Data are averages of real-time PCR results +/− std. error of the mean, n = 10. * indicates p<0.05 between groups.
(TIF)

Supporting Information S4 **Insulin-stimulated suppression of G6pc mRNA was inhibited by an Akt inhibitor (TCN).** Relative expression is relative to no insulin. The experiment was performed in control cells not exposed to palmitate. Data are averages of real-time PCR results +/− std. error of the mean, n = 6. * indicates p<0.05 between groups.
(TIF)

Supporting Information S5 **Palmitate-treatment did not affect Pten, α-Calcineurin and Phlpp1&2 protein levels in hepatocytes**. □ Control; ▪ 0.5 mM Palmitate. Protein levels were normalized to β-Actin. Data are averages of western blot quantifications +/− std. error of the mean, n = 6–8. * indicates p<0.05. Representative western blots are shown.
(TIF)

Supporting Information S6 **Metabolic parameters of experimental animals.** fa/fa rats were severely insulin-resistant, hyperglycaemic and hypertriglyceridemic, and had higher levels of plasma free fatty acids than their lean (Fa/Fa) counterparts. Prior to sacrifice a fasting blood sample was taken and plasma glucose, insulin, TGs and FFAs were determined. Data are averages of +/− std. deviation, n = 8 for fa/fa rats and n = 6 for Fa/Fa rats. * indicates p<0.05 between groups.
(DOC)

Supporting Information S7 **Akt-inactivating phosphatases are differentially regulated in diabetic ZDF rats.** α-Calcineurin (Ppp3ca) was significantly upregulated in fa/fa livers, while α-Calcineurin and Phlpp1 were significantly upregulated in fa/fa muscle compared to their lean Fa/Fa counterparts. Relative expression is relative to Fa/Fa expression levels. Data are averages of real-time PCR results +/− std. error of the mean. n = 8 for fa/fa rats and n = 6 for Fa/Fa rats. * indicates p<0.05.
(TIF)
