# Supplementary figures and images for: Free Fatty Acid-Induced PP2A Hyperactivity Selectively Impairs Hepatic Insulin Action on Glucose Metabolism
Source: PLoS One. 2011 Nov 7;6(11):e27424. doi: 10.1371/journal.pone.0027424 (PMC3210172; doi:10.1371/journal.pone.0027424)

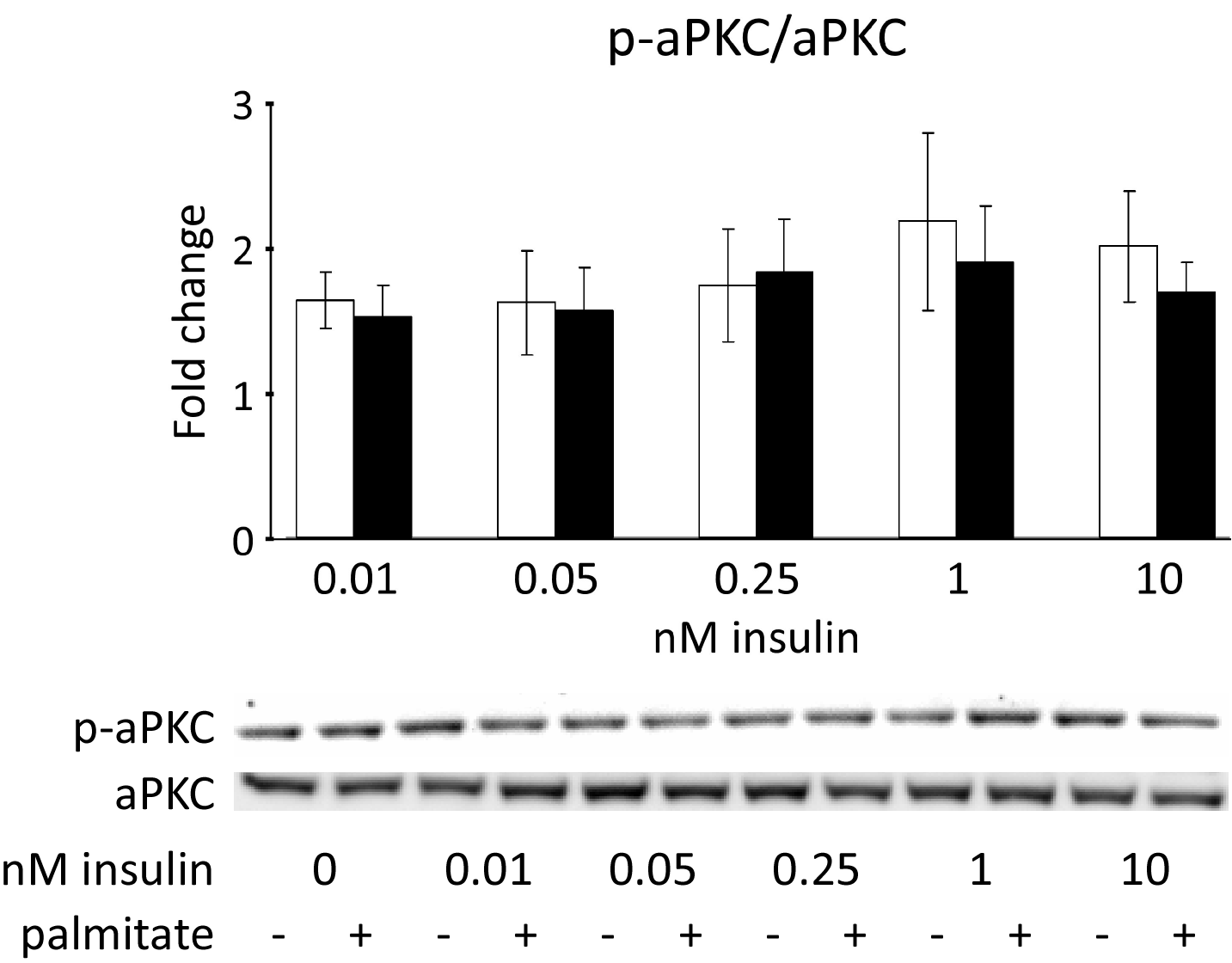

Supplement: Supporting Information S1 — Palmitate has no effect on insulin-stimulated aPKC phosphorylation in cultured primary rat hepatocytes. □ Control; ▪ 0.5 mM Palmitate. Fold change is relative to no insulin. The phosphorylation level was normalized to the total amounts of aPKC. Data are averages of western blot quantifications +/− std. error of the mean, n = 8. * indicates p<0.05 between groups. Representative western blot is shown. (TIF) [file pone.0027424.s001.tif]

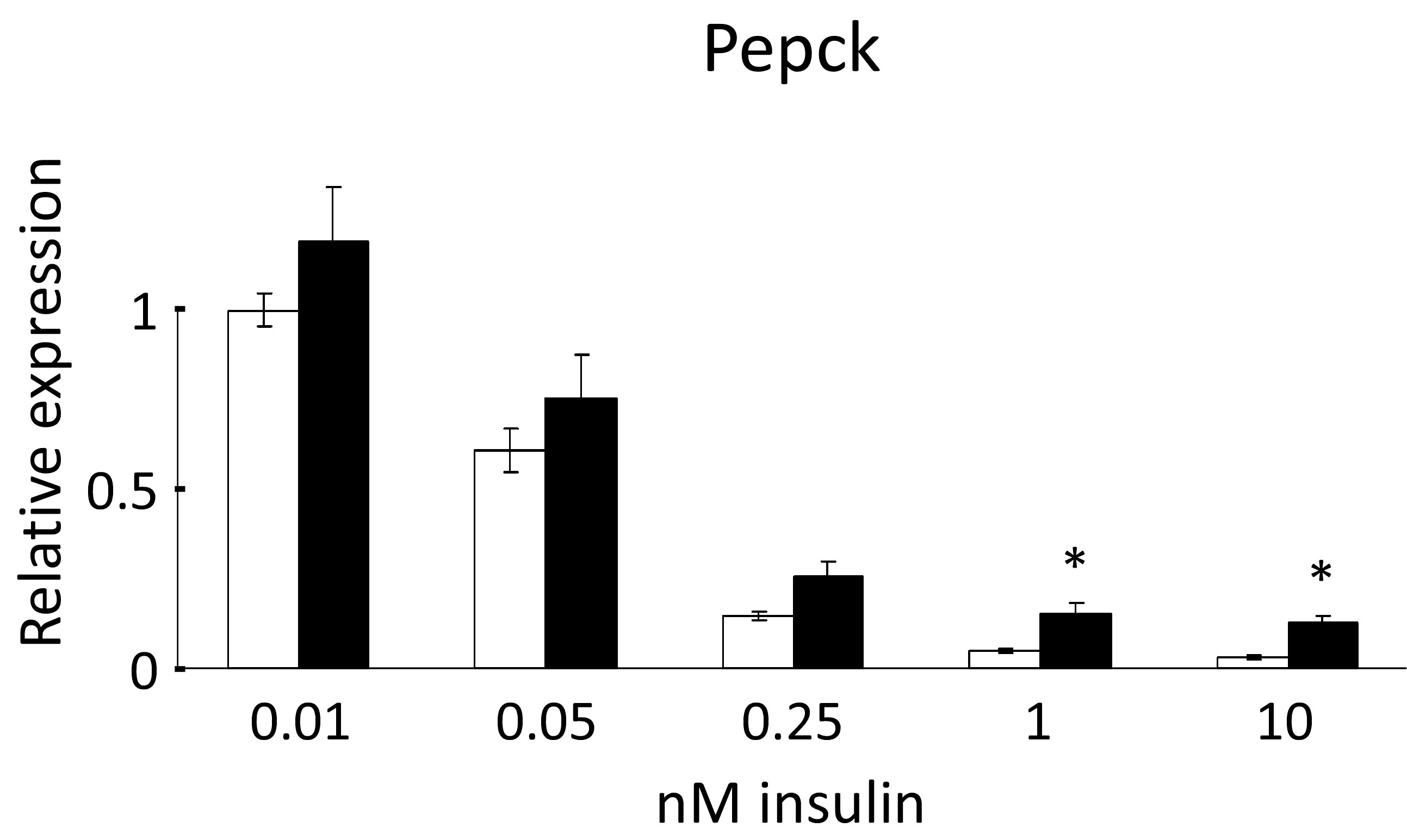

Supplement: Supporting Information S2 — Palmitate-induced insulin resistance is characterized by impaired Pepck suppression. □ Control; ▪ 0.5 mM Palmitate. Insulin-suppression of Pepck mRNA was significantly inhibited in hepatocytes treated for 20 h with palmitate in vitro. Relative expression is relative to no insulin. Data are averages of real-time PCR results +/− std. error of the mean, n = 10. * indicates p<0.05 between groups. (TIF) [file pone.0027424.s002.tif]

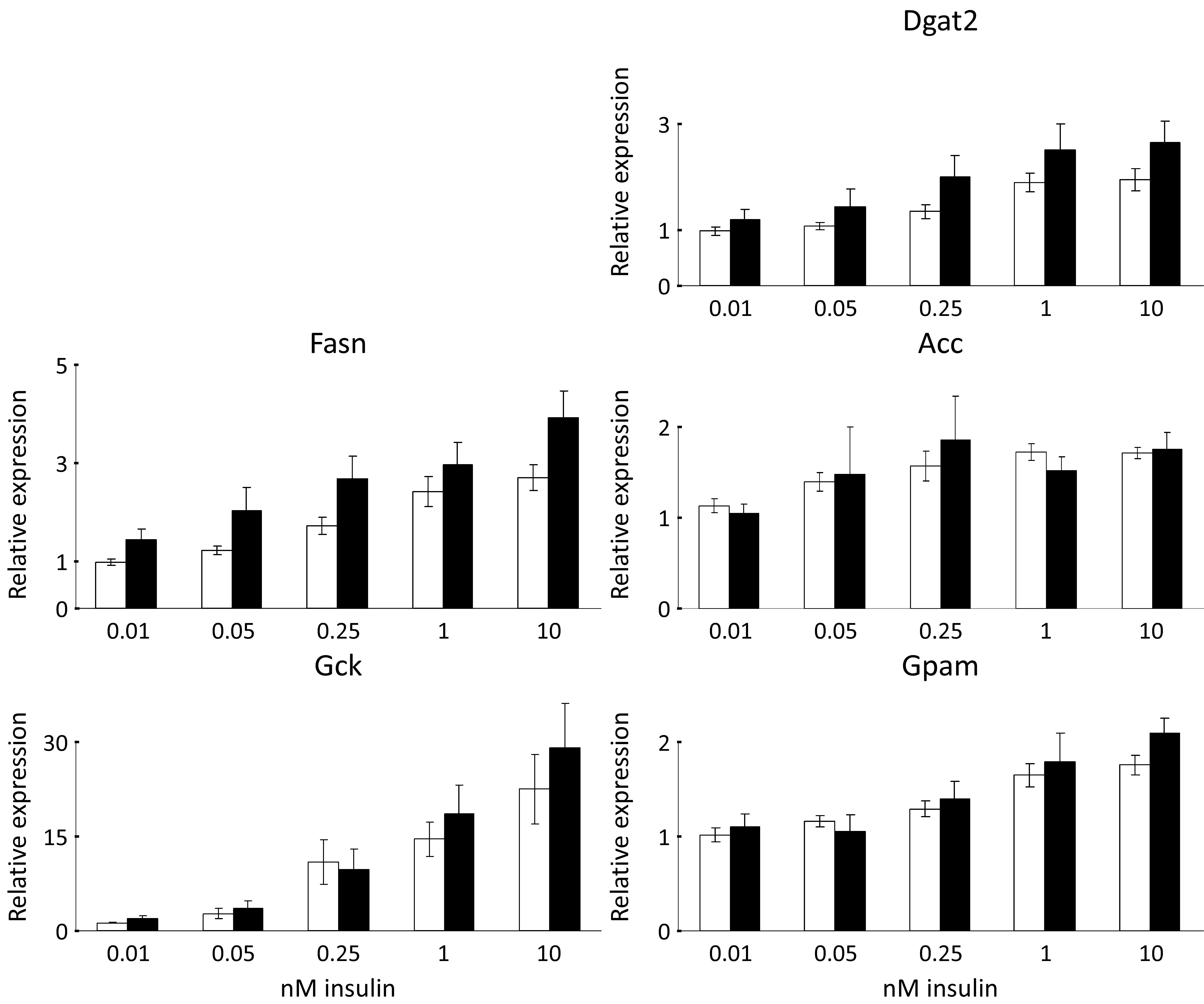

Supplement: Supporting Information S3 — Insulin-stimulated induction of lipogenic Srebp1c target genes was maintained in palmitate-treated cells. □ Control; ▪ 0.5 mM Palmitate. In contrast to the gluconeogenic genes, G6pc and Pepck, insulin maintained its effect upon Dgat2, Fasn, Acc, Gck and Gpam expression in insulin-resistant, palmitate-treated cells. Relative expression is relative to no insulin. Data are averages of real-time PCR results +/− std. error of the mean, n = 10. * indicates p<0.05 between groups. (TIF) [file pone.0027424.s003.tif]

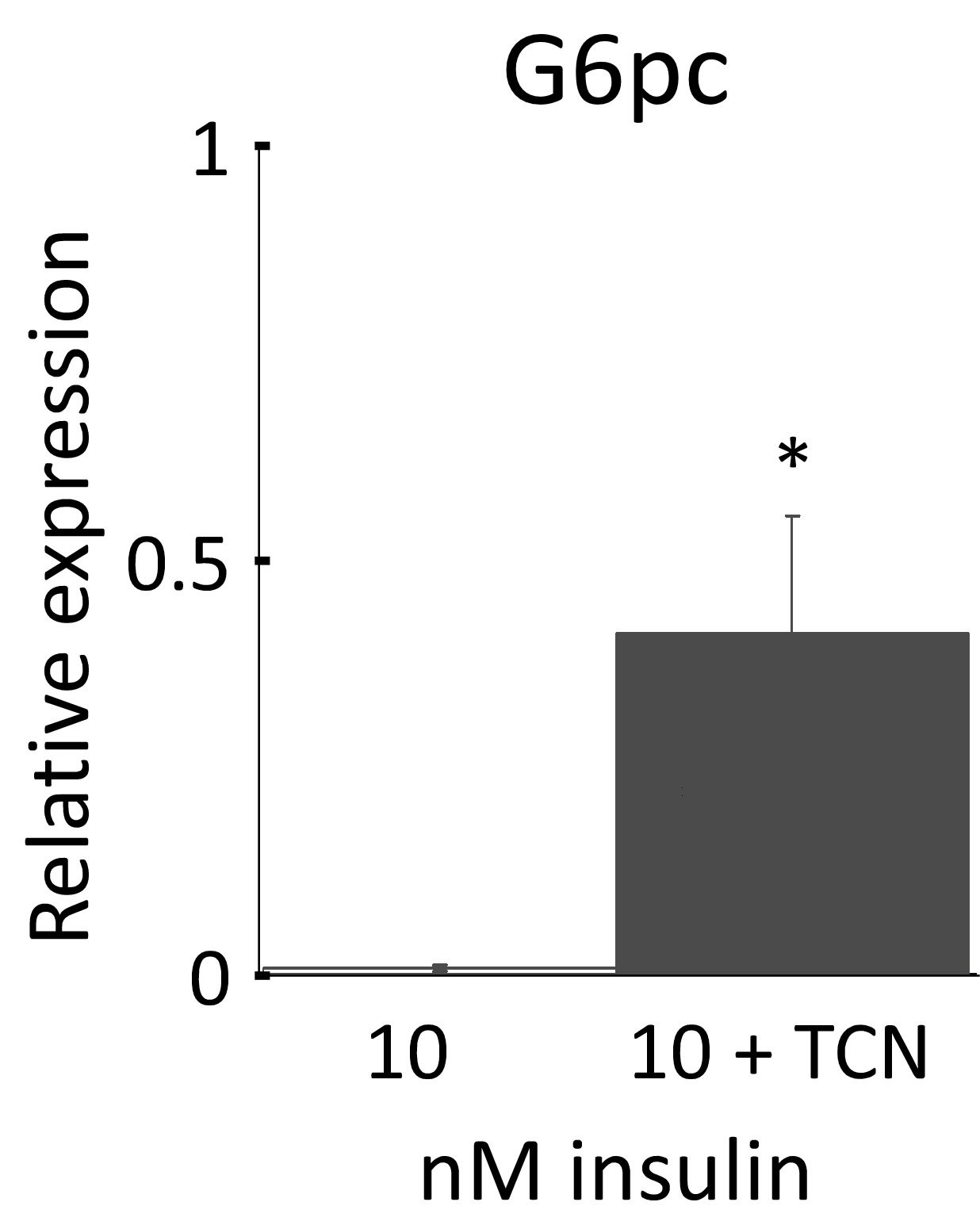

Supplement: Supporting Information S4 — Insulin-stimulated suppression of G6pc mRNA was inhibited by an Akt inhibitor (TCN). Relative expression is relative to no insulin. The experiment was performed in control cells not exposed to palmitate. Data are averages of real-time PCR results +/− std. error of the mean, n = 6. * indicates p<0.05 between groups. (TIF) [file pone.0027424.s004.tif]

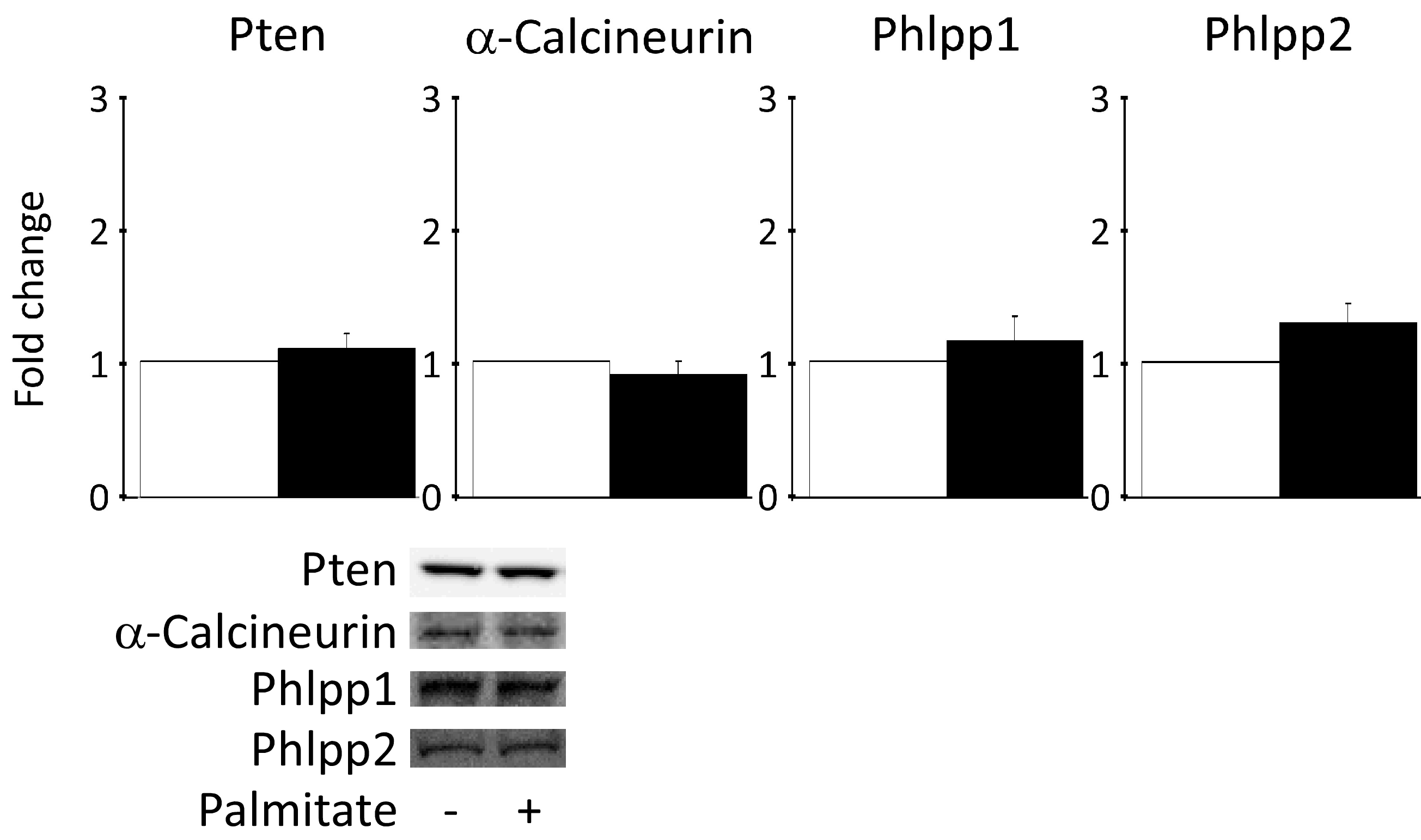

Supplement: Supporting Information S5 — Palmitate-treatment did not affect Pten, α-Calcineurin and Phlpp1&2 protein levels in hepatocytes. □ Control; ▪ 0.5 mM Palmitate. Protein levels were normalized to β-Actin. Data are averages of western blot quantifications +/− std. error of the mean, n = 6–8. * indicates p<0.05. Representative western blots are shown. (TIF) [file pone.0027424.s005.tif]

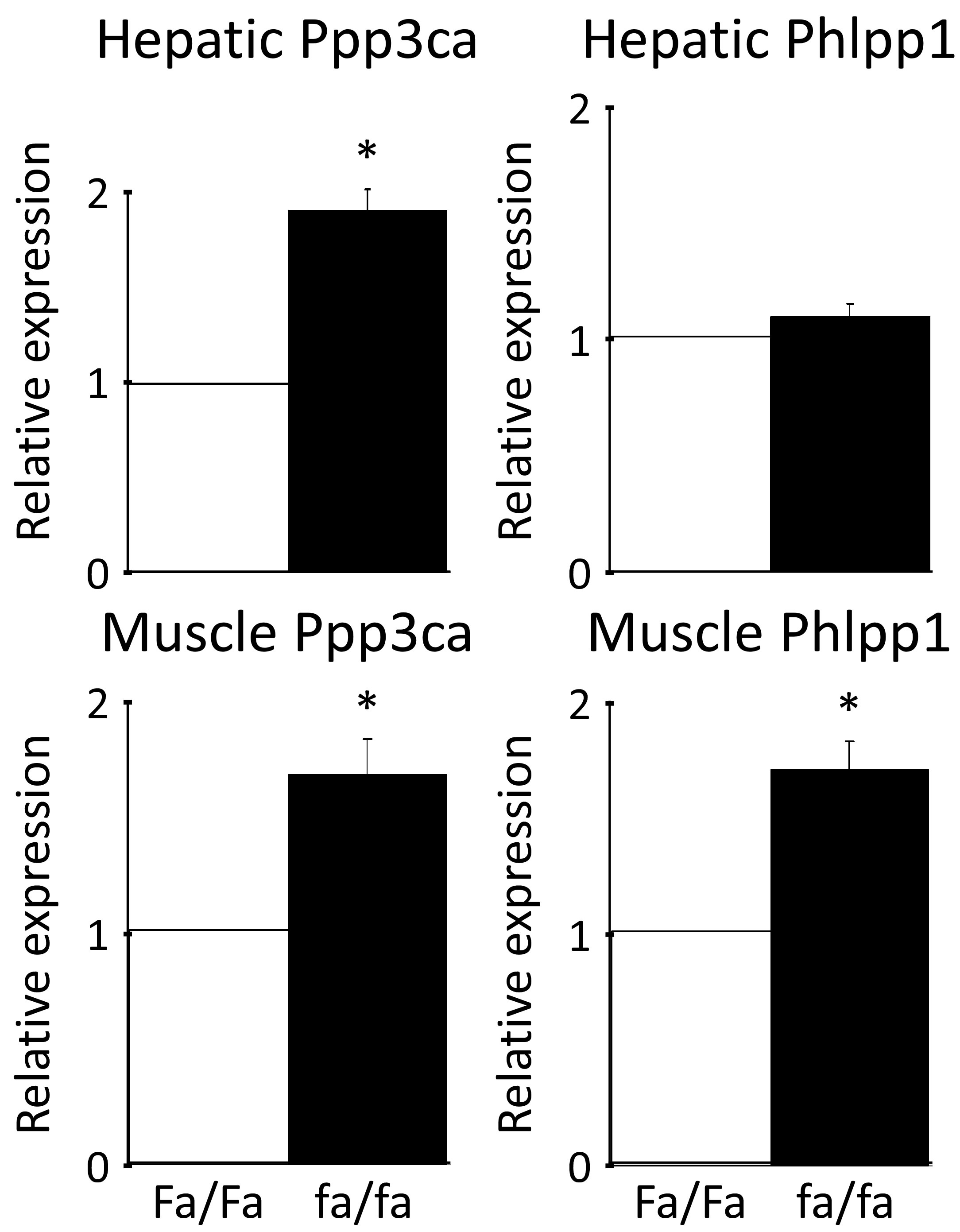

Supplement: Supporting Information S7 — Akt-inactivating phosphatases are differentially regulated in diabetic ZDF rats. α-Calcineurin (Ppp3ca) was significantly upregulated in fa/fa livers, while α-Calcineurin and Phlpp1 were significantly upregulated in fa/fa muscle compared to their lean Fa/Fa counterparts. Relative expression is relative to Fa/Fa expression levels. Data are averages of real-time PCR results +/− std. error of the mean. n = 8 for fa/fa rats and n = 6 for Fa/Fa rats. * indicates p<0.05. (TIF) [file pone.0027424.s007.tif]
