# Supplementary material for: Free Fatty Acid-Induced PP2A Hyperactivity Selectively Impairs Hepatic Insulin Action on Glucose Metabolism
Source: PLoS One. 2011 Nov 7;6(11):e27424. doi: 10.1371/journal.pone.0027424 (PMC3210172; doi:10.1371/journal.pone.0027424)
Supplement: Supporting Information S6 — Metabolic parameters of experimental animals. fa/fa rats were severely insulin-resistant, hyperglycaemic and hypertriglyceridemic, and had higher levels of plasma free fatty acids than their lean (Fa/Fa) counterparts. Prior to sacrifice a fasting blood sample was taken and plasma glucose, insulin, TGs and FFAs were determined. Data are averages of +/− std. deviation, n = 8 for fa/fa rats and n = 6 for Fa/Fa rats. * indicates p<0.05 between groups. (DOC) [file pone.0027424.s006.doc]

|  | **Zucker lean (Fa/Fa)** | **Zucker Obese (fa/fa)** |
| --- | --- | --- |
| Glucose (mM) | 10.0±0.2 | 13.1±1.0* |
| Insulin (mU/L) | 15.5±4.8 | 186.3±44.6* |
| Triglycerides (mM) | 0.37±0.03 | 6.10±0.45* |
| FFAs (mM) | 0.38±0.03 | 0.65±0.04* |
| Bodyweight (g) | 337±6 | 501±8* |
